# Preferences for Advance Directives in Korea

**DOI:** 10.1155/2012/873892

**Published:** 2011-09-08

**Authors:** So-Sun Kim, Won-Hee Lee, JooYoung Cheon, Jung-Eun Lee, KiSun Yeo, JuHee Lee

**Affiliations:** ^1^Nursing Policy Research Institute, College of Nursing, Yonsei University, Seoul 120-752, Republic of Korea; ^2^College of Nursing, Yonsei University, Seoul 120-752, Republic of Korea

## Abstract

*Background*. The goal of advance directives is to help patients retain their dignity and autonomy by making their own decisions regarding end-stage medical treatment. The purpose of this study was to examine preferences of advance directives among general population in Korea. *Method*. A descriptive cross-sectional survey was performed from October 2007 to June 2008 in Seoul, Korea. A total of 336 city-dwelling adults self-administered the questionnaire and returned it via mail. Data analyses were conducted using SPSS 17.0. *Results*. Subjects reported the need for healthcare providers' detailed explanations and recommendations regarding end-of-life care. When there is no hope of recovery and death is imminent, most subjects did not want to receive cardiopulmonary resuscitation nor an IV or tube feeding. However, most of the subjects wanted pain management care. *Conclusions*. The present study showed that many Korean people have an interest in advance directives. The results show that the autonomy and dignity of patient have increased in importance. To provide better end-of-life care, there is a need to educate patients on the definition and intent of an advance directive. Additional proactive communication between patients and their caregivers should be educated to healthcare providers.

## 1. Background

An advance directive (AD) is an “oral or written statement in which people declare their treatment preferences in the event that they lose decision-making capacity” [1]. ADs are used to enhance the autonomy of patients for when they are unable to make medical decisions or express their preferences by themselves [2]. Therefore, ADs become a kind of a truism to assert respect for the patient in regard to their autonomy [3]. Capron [4] has stated that when a patient has a properly executed AD, the patient would, in theory, alleviate much of the uncertainty that often paralyzes physicians and family members (or other surrogate decision makers) and would facilitate the resolution that best reflects their true wishes regarding their care [5].

In the USA, ADs have received widespread attention ever since the enactment of the Patient Self-Determination Act (PSDA, Public Law no. 101–508) in 1991 [6]. Various laws related to ADs give patients the right to express their wishes for end-of-life situations. The PSDA ensures that advance care planning is documented in the patient's medical record in advance and makes it as a formal document. The estimates of those with ADs vary from 16 to 26% in the USA [7]. Healthcare providers consistently try to apply ADs to older patients or terminal patients that are in a clinical setting as an attempt to improve the quality of end-of-life care by communicating with both the patients and their family members.

In Korea, with more than 24,600 deaths annually, the percentage of deaths that occur in hospitals increased from 28.5% in 1998 to 63.7% in 2008 [8]. Culturally, Korean people generally feel uncomfortable talking about death and have traditionally made medical decisions based on what is best for the family rather than allowing the patient to decide for themselves [9]. In other words, most of the patients in Korea have little opportunity to prepare ADs [10]. A recent survey has shown that 84% of the decisions made in regard to ADs are made by physicians [11]; however, another survey has found that AD decisions are mostly made by the offspring or spouse of the patient [12–14]. 

However, there is an increasing interest in death and of end-of-life care as a result of our increased life span. A survey of cancer patients in Korea has shown that 96.1% of patients want to be made aware of their (end-of-life) condition. However, only 78.3% of family members want to inform the patients of their condition [15]. Thus, a conflict exits between the opinions of the patients and their families. 

Korea had its first legal exposure to end-of-life issues in 1997 with the Boramae Hospital case [5]. In this case, physicians were unable to persuade the patient's wife to maintain the required therapies and were later charged and found guilty of discharging their postoperative patient after discontinuing the life-sustaining therapy. Prior to this case, physicians in Korea had been able to withdraw life-sustaining therapy with the consent of the patient's delegate [16]. As recently as May 2009, the Korean Supreme Court recognized the validity of ADs as a result of the nation's first civil case concerning the withdrawal of life-sustaining treatment for an incompetent patient [5]. The legislation of ADs regarding removal of life-sustaining devices from comatose patients that have no hope of resuscitation has been dealt with in the Supreme Court. In regard to the issue of stopping meaningless end-of-life treatments in terminally ill patients, the National Evidence-based Healthcare Collaborating Agency (NECA) of Korea held three consecutive consensus meetings in July 2009. The contents of these meetings were stated as follows. (1) Basic care such as fluids, nutritional support, and pain control should be maintained. (2) When a terminally ill patient expresses his or her wishes to refuse cardiopulmonary resuscitation (CPR) or ventilator support, the CPR or the ventilation care can be stopped. (3) The patient can express their wishes regarding life-sustaining treatments other than cardiopulmonary resuscitation or ventilator support. (4) The physician should take the patient's wishes into consideration when making a medical decision. (5) Euthanasia and physician-assisted suicide are unacceptable [17]. 

As social interests regarding ADs increase, a few researchers have examined AD issues in Korea [18–21]. A survey of adults and healthcare providers shows that 78.8% of participants express interest in ADs [22]. However, cultural beliefs or values can have differing impacts on the attitudes regarding ADs among people in Korea [23–25]. Since death is not a topic that is readily discussed or expressed, people may believe that it is wrong to refuse medical treatment at the end-of-life stage. Furthermore, there have only been a few studies that have examined the attitudes or the perceptions of death, which has resulted in a limited view of ADs in this population. 

By providing an AD in a document, patients are able to make their wishes known as well as provide both clarity and comfort to the family members [26]. With this view of ADs, this study aimed to examine how ADs were perceived in the general population of Korea in order to ultimately stimulate future research in this area.

## 2. Methods

### 2.1. Design

A descriptive cross-sectional survey was performed to investigate the attitudes and perceptions of people in Korea regarding ADs. Data were collected using convenience sampling from Oct 2007 to June 2008 using a mailed survey. Permission from the Institutional Review Board (IRB) was obtained prior to data collection from university setting. In order to meet ethical considerations, participants received a document explaining of anonymity, confidentiality, rights to withdraw freely, and benefit/risk of completing the questionnaire. Informed consent was obtained from each participant.

### 2.2. Sample and Data Collection

Participants in this study were adults (age ≥18 years) residents of Seoul, Korea who had agree to participate. In order to conduct a representative survey, Moser and Kalton's formula [27] was used to determine the sample size and the acceptable margin of error was set at three percent (0.03) for this study. According to this formula, given that the sampling population (people preferred to get palliative care) was about 85% in Korea [28], a sample size of at least 141 participants were necessary to give significant results.

We first contacted the heads of public offices, companies, and resident associations in Seoul. After receiving approval from these people, we then mailed the study materials and a formal consent form to all of the associated members. One public office, one company, and two resident associations were included in this study. All of the participants provided signed informed consent. Only eligible patients who agreed to participate in the study were included. Among them, 336 subjects self-administered the questionnaire and returned it to us by mail. A small incentive was provided at the time of initial contact in order to increase the completion rate.

### 2.3. Questionnaire

A questionnaire was developed and validated by Miyata et al. [29], in which respondents were asked about their preferences and attitudes regarding ADs. Respondents were also asked about their preferences regarding treatment, which were translated from actual living will declarations from people in the USA. The original questionnaire items were as follows: “If my condition is determined to be terminal and incurable, I do want life-sustaining procedures that serve only to prolong the process of my dying,” “If I am in an irreversible or incurable persistent vegetative state, I do want cardiac resuscitation,” “If I am in an irreversible or incurable persistent vegetative state, I do want artificial nutrition and hydration,” and “I want my life to be prolonged to the greatest extent possible.” The questionnaire asked respondents to what extent they wanted to express their preferences regarding treatment, how they would like to discuss treatment preferences with their proxy, and to what extent their proxy should respect their preferences regarding treatment [29].

Since the questionnaire is in Japanese and no Korean or English version existed for use in our study, we received permission from the author to translate the questionnaire from Japanese into Korean. Two bilingual (Japanese and Korean) native Korean-speaking nursing professionals translated the questionnaire into Korean. Any Japanese phrases that were difficult for them to understand were translated after consensus. Two other bilingual native Japanese-speaking translators then performed the back translations of the first Korean version. Any differences between the back-translated version and the original version were further discussed, and applicable modifications were made. 

To test the item clarity and content validity, the translated version was submitted to five nursing professionals that were familiar with the subject matter referenced in the questionnaire. The content validity of the questionnaire was assessed by calculating the content validity index (CVI) [30]. The overall CVI was 0.90, which indicated satisfactory agreement of the Korean version among the professionals. The translated version was then tested on a pilot group that consisted of 20 adults. After they had completed the questionnaire, they were asked if the translated questions were easy to understand and their responses were used to determine that no further revisions were required. 

### 2.4. Data Analysis

Data were entered into SPSS 17.0, and an investigator confirmed the accuracy of the data entry by comparing the answers with those in the original questionnaires. Descriptive analyses were conducted to measure the sociodemographic characteristics and preferences for ADs. Chi-square tests were performed to assess the differences in the preferences for end-of-life treatment according to sociodemographic characteristics.

## 3. Findings

Table 1 presents the sociodemographic characteristics of the study participants. A total of 336 subjects completed and returned their questionnaires (44.3% were men). The mean age of the participants was 43 years with a SD of 9.5 (range 18–74 years). Many of the subjects (*n *= 199, 59.2%) had a greater than university level of education, and many rated their health as neutral (*n *= 181, 53.9%). Only 31% (*n *= 104) of subjects answered that they were in good or very good health. Approximately half of the participants claimed their socioeconomic status to be neutral (*n *= 178, 53%), and approximately 33% of respondents did not affiliate with any religion. More than half of the subjects had experienced the death of a loved one (*n *= 241, 71.7%). More than 50% of the subjects reported that they had not ever given thought to their end-stage treatment desires (*n *= 196, 58.3%), and most of the subjects did not have a will (*n *= 279, 83%). 

Subjects (*n* = 125, 37%) reported the need for an explanation of ADs from their healthcare provider. Additionally, they expressed desire for AD recommendation from their healthcare provider if they were ever to be in an advanced disease state (*n* = 205, 61%).  Table 2 presents the preferences for end-of-life treatment under specific circumstances. More than 60% (*n* = 213) of the subjects reported that they did not want cardiopulmonary resuscitation if they were ever to be at the end-of-life stage without any hope of recovery. The majority of subjects did not want to have an IV or tube feeding (*n* = 228, 67.9%). However, if there is no hope of any recovery and death is imminent, most of the study subjects wanted to receive pain management (*n* = 200, 59.5%) while 64.6% (*n* = 217) of the subjects did not want any type of aggressive life-sustaining treatment. Interestingly, 8.9% (*n* = 30) wanted to receive active medical treatment regardless of their health status while 60.7% (*n* = 204) did not. Additionally, in cases in which subjects cannot make their own decisions regarding treatment, 40% (*n* = 134) of the subjects wanted their proxy and doctor decide on the care plan while 28% (*n* = 95) of the subjects wanted the proxy alone to make a care decisions.

Table 3 lists the attitudes of subjects toward ADs. The results show that 69.3% (*n* = 233) of the participants reported that they would verbally express their medical preferences, while 60.1% (*n* = 202) of the subjects wanted a written document to state these preferences. Forty percent of the subjects (*n* = 132) wanted to express their treatment preferences generally rather than concretely. Most of the subjects (*n* = 288, 85.7%) wanted direct notification of disease in cases of cancer or chronic disease. More than 80% of the subjects did not have a proxy (*n* = 277); however, many expressed the desire to obtain such an agent to make medical treatment decisions (*n* = 237). 

Chi-square analysis showed that only education status was identified as being significantly associated with the preferences for end-of-life treatment (Table 4). The participants that had a higher level of education were more likely to want pain management even though the treatment may reduce their life expectancy (*x^2^* = 14.296, *P* < 0.001). Education level was categorized into two groups: low level (middle or high school graduation) and high level (college or university graduation). Other sociodemographic characteristics such as age, gender, self-reported health status, socioeconomic status, and religion were not significantly associated with treatment preferences.

Table 5 shows that most participants indentified their spouse as their first choice as their proxy regarding medical treatment (*n* = 243, 72.3%). Subjects also wanted to be respected by their proxy regarding their treatment preferences (*n* = 260, 77.4%). In regard to treatment preferences, most respondents expressed the desire for multiple conversations with their respective proxy regarding treatment preferences (*n* = 235, 70%), especially in the case of advanced disease. If the proxy has a different opinion than does the patient's physician, then many subjects preferred the opinion of the physician (*n* = 174, 51.4%). 

## 4. Discussion

With the rapid increase in the number of older people in Korea, health professionals and researchers should have a better understanding of the preferences and attitudes of the general population in regard to ADs. The present study examined the understanding of ADs among Koreans. The findings suggested that subjects acknowledge the importance of patient autonomy and dignity when deciding on end-of-life care. More than half of the subjects wanted pain management as an end-of-life treatment, but they did not want aggressive medical treatments such as CPR, IV, or tube feeding. The majority of participants would like to verbally express their directives rather than use a written document. There was a strong desire for a proxy agent, and the most preferred proxy was the patient's spouse. 

Caution should be exercised when interpreting the results of this study since a convenience sampling method was used in an urban area. Most of the participants were well educated and had relatively high socioeconomic status, which may have had an impact on the results. Previous studies have demonstrated that higher levels of education are associated with more positive attitudes regarding end-of-life care and communication [31, 32]. Furthermore, this study was a cross-sectional survey of people living in a metropolitan area in Korea and the results may not be applicable to populations in other countries. 

Overall, the results of this study were similar with those of previous reports. First, it was not surprising that most of subjects had not thought about issues pertaining to ADs as Asians generally do not feel comfortable dealing with end-of-life issues [33–35]. However, most participants expressed the desire for an explanation of ADs if they should find themselves in a terminal disease state. Therefore, discussion of end-of-life care between patients and healthcare professionals is needed in order for there to be a better understanding of ADs in the clinical setting. 

Study participants wanted to communicate with their healthcare providers to obtain knowledge regarding ADs. Furthermore, they wanted to verbally express their directives rather than use written documentation. The most common method for establishing healthcare preferences can be achieved with an informal conversation [36, 37]. Preferences for the verbal type of directives were similar to those reported in a Japanese study. However, the use of a written document is suggested over oral ADs since such verbal directives are usually forgotten or misinterpreted or may not be directly reported to healthcare providers [33]. 

In addition, the results of the present study showed that when subjects need to make an end-of-life decision, the involvement of family or a physician was desired. Most of the participants chose their spouse as their proxy decision maker. Culturally, Asian people tend to value the opinions of their family members and that of the healthcare provider over their own personal opinion [9, 29, 35, 38] and this was reflected in the findings of the present study. Cultural values and health beliefs both have an impact on end-of-life decisions. For example, African Americans prefer to use life-sustaining methods while Asians and Hispanics focus more on what is best for the family as a whole. One reason for these cultural differences may be due to limited communication with healthcare providers, which may result in inadequate trust or a personal bias when writing down the wishes of their patients [38]. Therefore, more culturally sensitive and specific nursing care plans are warranted in this area [34, 39]. 

The findings of one study indicated that patient autonomy was considered important in discussions of treatment decisions [40]. Frequently, healthcare providers have used the term ADs along with DNR (do not resuscitate) when focusing on CPR status and, thus, there has been a limited provision of in-depth discussion in advanced care planning [36]. We believe that benefits of ADs are more satisfied with when patients received appropriate care. Healthcare providers should improve the care they provide to both patients and families by having a better understanding of the patient needs and experiences in regard to end-of-life treatment. 

This study provided that many people in Korea have an interest in ADs, that there is a need for more education regarding ADs. There is a need for better communication between patients and caregivers to improve the end-of-life care. Future studies are needed to further examine the attitudes and preferences of healthcare providers regarding ADs compared to those of patients and caregivers.

## Figures and Tables

**Table 1:** Sociodemographic characteristics (*N *= 336).

Variable	Frequency (*n*)	Percentage (%)
Age		
≤30 years old	35	10.4
31–40	93	27.7
41–50	152	45.2
51–60	45	13.4
≥61 years old	11	3.3

Gender		
Male	149	44.3
Female	187	55.7

Education		
Middle school	18	5.4
High school	92	27.4
College	22	6.5
≥university	199	59.2
Other	5	1.5

Self-reported health status		
Very good	59	17.6
Good	45	13.4
Neutral	181	53.9
Bad	48	14.3
Very bad	3	0.9

Socioeconomic status		
Fairly good	87	25.9
Neutral	178	53
Fairly bad	71	21.1

Religion		
None	112	33.3
Buddhism	54	16.1
Catholicism	33	9.8
Christianity	132	39.3
Other	5	1.5

Past experiences of separation by death		
Yes	241	71.7
No	95	28.3

Having a will		
Yes	57	17
No	279	83

Total	336	100

**Table 2:** Preferences for end-of-life treatment (*N* = 336).

	Yes (%)	I do not know (%)	No (%)
If there is no hope of my recovery and the death is drawing near, I want to be treated for relieving pain although that treatment can reduce my life (Preference item 1)	200 (59.5)	64 (19)	72 (19.2)
If there is no hope of my recovery and the death is near, I want to be treated aggressively to extend or sustain my life even with no effect for recovery (Preference item 2)	34 (10.1)	85 (25.3)	217 (64.6)
If I am living as a vegetarian, I want to be given cardiopulmonary resuscitation using defibrillator (Preference item 3)	54 (16.1)	69 (20.5)	213 (63.4)
If I am living as a vegetarian, I want to be given IV therapy and nasogastric tube feeding (Preference item 4)	23 (6.8)	85 (25.3)	228 (67.9)
I want to be treated to extend my life in any case (Preference item 5)	30 (8.9)	102 (30.4)	204 (60.7)

**Table 3:** Attitudes regarding advance directives (*N *= 336).

	Agree a lot (%)	Agree a little (%)	Neither agree nor disagree (%)	Disagree a little (%)	Disagree a lot (%)
I want to prepare a verbal promise indicating medical treatment preference if needed	84 (25)	149 (44.3)	68 (20.2)	28 (8.3)	7 (2.1)

I want to prepare a document describing medical treatment preference if needed	90 (26.8)	112 (33.3)	82 (24.4)	39 (11.6)	13 (3.9)

I want to have a power of attorney for possible medical treatment	113 (33.6)	124 (36.9)	64 (19)	26 (7.7)	9 (2.7)

I want to propose whether someone notify me if I have a cancer or chronic illness	167 (49.7)	121 (36)	29 (8.6)	16 (4.8)	3 (0.9)

**Table 4:** Preferences for end-of-life treatment by sociodemographic characteristics (*N *= 336).

Characteristics	Categories	P1		P2		P3		P4		P5	
		%*	*x^2^*	%	*x^2^*	%	*x^2^*	%	*x^2^*	%	*x^2^*
Age	≤40	60.2	0.122 (*P* = .941)	12.5	2.390 (*P* = .303)	21.9	6.038 (*P* = .050)	7.0	0.263 (*P* = .958)	10.2	1.803 (*P* = .407)
	41-40	58.5		9.1		13.3		7.7		6.3	
	≥51	60.7		5.4		9.1		5.4		10.7	

Gender	Male	62.4	0.946 (*P* = .331)	9.4	0.018 (*P* = .892)	19.5	2.883 (*P* = .090)	5.4	1.018 (*P* = .313)	10.7	0.922 (*P* = .337)
	Female	57.1		9.8		12.6		8.2		7.7	

Education	≤high school	45.2	14.296 (*P* = .000)	9.6	0.087 (*P* = .768)	12.3	2.072 (*P* = .150)	7.8	0.220 (*P* = .639)	5.2	3.122 (*P* = .077)
	>college	66.7		10.6		18.4		6.5		11.1	

Self-reported health status	Good	59.2	2.322 (*P* = .313)	8.7	0.451 (*P* = .798)	21.2	2.839 (*P* = .242)	7.7	0.202 (*P* = .956)	7.7	0.384 (*P* = .846)
	Neutral	56.7		10.7		13.5		6.7		9.6	
	Bad	68.6		11.8		16.0		5.9		9.8	

Socioeconomic status	Fairly good	67.8	3.839 (*P* = .147)	5.7	2.645 (*P* = .267)	14.9	0.163 (*P* = .922)	4.6	1.076 (*P* = .577)	9.3	0.666 (*P* = .717)
	Neutral	57.5		11.4		16.6		7.4		8.0	
	Fairly bad	53.5		12.7		17.1		8.5		11.3	

Religion	None	58.6	1.065 (*P* = .925)	6.3	4.803 (*P* = .274)	15.2	3.646 (*P* = .427)	7.1	1.915 (*P* = .731)	9.0	0.704 (*P* = .935)
	Buddhism	61.1		16.7		17.0		11.1		11.1	
	Catholicism	66.7		12.1		9.1		6.1		9.1	
	Christianity	57.6		11.2		17.6		5.6		8.0	
	Other	60.0		0		40.0		0		0	

Past experiences of separation by death	Yes	61.1	1.084 (*P* = .321)	10.9	0.413 (*P* = .521)	16.4	0.009 (*P* = .924)	6.3	0.524 (*P* = .469)	9.7	0.403 (*P* = .526)
	No	54.8		8.5		16.0		8.5		7.4	

P1: Preference item 1, P2: Preference item 2, P3: Preference item 3, P4: Preference item 4, and P5: Preference item 5.

*Each percent means how many participants chose yes for that item.

**Table 5:** Preferences for possible power of attorney in end-of-life care (*N = *336).

	Frequency (*n*)	Percentage (%)
Spouse	243	72.3
Parents	28	8.3
Adult children	18	5.4
Sibling	12	3.6
Relatives except parents, adult children, and sibling	9	2.7
Doctor	22	6.5
Lawyer	2	0.6

Total	336	100
